# Octenidine effectively reduces *Candida auris* colonisation on human skin

**DOI:** 10.1038/s41598-025-11914-x

**Published:** 2025-07-25

**Authors:** Diana Cerbu, Saskia Seiser, Trinh Phan-Canh, Doris Moser, Christian Freystätter, Johannes Matiasek, Karl Kuchler, Adelheid Elbe-Bürger

**Affiliations:** 1 Department of Dermatology, Medical University of Vienna, Währinger Gürtel 18-20, 1090 Vienna, Austria; 2 Max Perutz Labs Vienna, Medical University of Vienna, Campus Vienna Biocenter, Dr. Bohr-Gasse 9/2, 1030 Vienna, Austria; 3 Department of Cranio-Maxillofacial and Oral Surgery, Medical University of Vienna, Währinger Gürtel 18-20, 1090 Vienna, Austria; 4 Department of Plastic and Reconstructive Surgery, Medical University of Vienna, Währinger Gürtel 18-20, 1090 Vienna, Austria; 5 Plastic, Reconstructive and Aesthetic Surgery, Medizin Am Kärtner Ring 14/13, 1010 Vienna, Austria

**Keywords:** Antiseptics, Human skin, Ex vivo model, *Candidozymaauris* (formerly *Candida auris*), Antifungal stewardship, Antimicrobials, Clinical microbiology, Fungi

## Abstract

*Candidozyma* (formerly *Candida*) *auris* (*C. auris*), a WHO critical priority pathogen known for its multi-drug resistance and strong skin tropism, is posing a significant health threat. This study evaluates the efficacy of commercial octenidine-based antiseptics in reducing *C. auris* colonisation on intact and wounded human skin. Using an established ex vivo human skin model to simulate clinical settings, skin samples from healthy donors were exposed to planktonic *C. auris* cells. Six hours post-contamination, two ready-to-use octenidine-based antiseptics were applied, and fungal colonisation was assessed after 18 h via periodic acid-Schiff staining, bright field and scanning electron microscopy and colony forming unit quantification. In vitro biofilm assays with various *C. auris* strains, including drug resistant ones, were performed to determine the antifungal effects of octenidine formulations. Results showed that octenidine-based antiseptics significantly reduced *C. auris* viability on intact and wounded human skin, and also demonstrated a nearly complete eradication across tested strains in vitro. These findings highlight the potential of octenidine-based products in reducing *C. auris* colonisation, supporting infection prevention and control strategies in healthcare settings and enhancing patient safety.

## Introduction

Pathogenic fungi cause a wide range of infections, from skin and chronic mucocutaneous diseases to severe, life-threatening infections^1,2^. Of particular concern is the skin-tropic *C. auris*, first identified in Japan in 2009^3,4^, which has now spread globally causing outbreaks in healthcare settings. Systemic *C. auris* infections can lead to severe complications, including sepsis, multi-organ failure involving the kidney, heart, lung, brain, liver, and spleen, which may result in death, particularly in critically-ill patients^5^. The WHO and the CDC classified *C. auris* as a top priority fungal pathogen that requires immediate attention in research and drug discovery^6,7^. The six known *C. auris* clades^8^ show pronounced adhesion to inanimate but also living surfaces such as human skin where it can persist for extended periods^9^, which fosters nosocomial outbreaks^10–18^. Rapid identification is essential for effective therapy and implementation of appropriate infection control^19,20^. Although *C*. *auris* shows lower virulence than other Candida species^21,22^, it has acquired remarkable abilities to thrive in nutrient-limited environments and carries significant resistance to limited antifungal agents, such as azoles, amphotericin B, echinocandins as well as flucytosine^4,23–25^.

*C. auris* typically appears in a round-to-ovoid yeast morphology, but it can undergo phenotypic transitions, forming multicellular aggregates and pseudohyphae^26,27^. Its ability to form biofilms^23,28–30^ provides a protective environment that enhances its resistance to both antimicrobial treatments and host immune responses, complicating infection control^31,32^. Indeed, a key factor in catheter-associated Candidemia is the propensity of *C. auris* to colonise skin^28,33^. *C. auris* invades superficial skin niches without having to breach the epidermal barrier, but can reach deeper compartments through wounding^34^. Thereby, pseudohyphal forms extend into the upper dermis, creating biofilm-like layers^28,30,34^.

As microbial skin colonisation poses a significant risk for subsequent infection, especially in individuals with comorbidities^35^, whole-body decolonisation has been implemented in many healthcare facilities. Chlorhexidine (CHG) is the most frequently used substance for this indication, however it is associated with limited efficacy against *C. auris* in clinical settings, resulting in persistent skin colonisation^18,36^. Furthermore, CHG is occasionally associated with severe allergic and anaphylactic reactions^37,38^, and the meanwhile frequently described manifestations of reduced susceptibility to this antiseptic, including even cross-resistance to antibiotics (e.g. colistin) and antifungals (e.g. azoles), raises concerns among healthcare professionals about its use in infection control^39–44^.

Octenidine (OCT) is another synthetic antimicrobial molecule that has been successfully deployed for more than 35 years in Europe and later also in Australia and Asian countries for skin, wound and mucous membrane antisepsis^45–50^, treatment of vaginal infections^51–54^, or decolonisation of patients^55–60^. Besides its advantageous safety profile^61^, OCT is associated with a remanence effect of at least 48 h^50^, as the active ingredient is not absorbed through the skin. Any development of resistance to OCT has not yet been observed^40,43,62–66^ most probably due to its rapid and unspecific mode of action based on purely physical interactions with lipid membranes of different clinically relevant bacterial and fungal pathogens^67–71^. Moreover, OCT is a potent antiseptic with high in vitro efficacy even against drug-resistant pathogens like *C. albicans*, *C. auris* and *Nakaseomyces glabratus (C. glabrata)*^66,71–74^, and in contrast to triclosan or CHG, selection pressure upon long-term exposure to OCT did not induce cross-resistance to antifungals in Candida species^43^. OCT inhibits hyphal growth and biofilm formation, impairs ergosterol biosynthesis and triggers reactive oxygen species that compromise fungal cell membranes^71^. To assess whether commercially available OCT-based pharmaceuticals, octenisept (OCS, aqueous wound and mucous membrane antiseptic)^75^ and octeniderm (OCD, alcoholic skin antiseptic)^76^, can affect *C. auris* skin colonisation, we tested the products using a recently established ex vivo human skin model that can simulate clinical conditions with or without intact barriers^34,77^.

## Materials and methods

### *C*.* auris* growth and culture conditions

Representative clinical *C. auris* strains from different clades were used in this study, including 1133/P/13R and B8441, clade I; B11222, clade III; as well as B11245, clade IV^14,78^ (provided by Anurhada Chowdhary, Medical Mycology Unit, Vallabhbhai Patel Chest Institute, India). Fungal strains were cultured in rich YPD media containing 2% glucose (w/v) and 1% bacto yeast extract (Formedium, UK)^79^. *C. auris* was routinely grown at 30 °C with aeration in glass flasks in a rotary shaker at 200 × revolutions per minute (rpm) (Eppendorf Innova 44 Incubator Shaker, Thermo Fisher Scientific, USA) to the exponential growth phase. Cultures were harvested by a short spin at 3000xg (Eppendorf Centrifuge 5427R, Merck KGaA, Germany), and washed once with phosphate-buffered saline (PBS; Thermo Fisher Scientific)^79^. Cell numbers were quantified (Casy TTC Cell Counter, Roche, Switzerland) and adjusted to the required cell density with PBS.

### Ex vivo human skin infection models

**Fig. 1:**
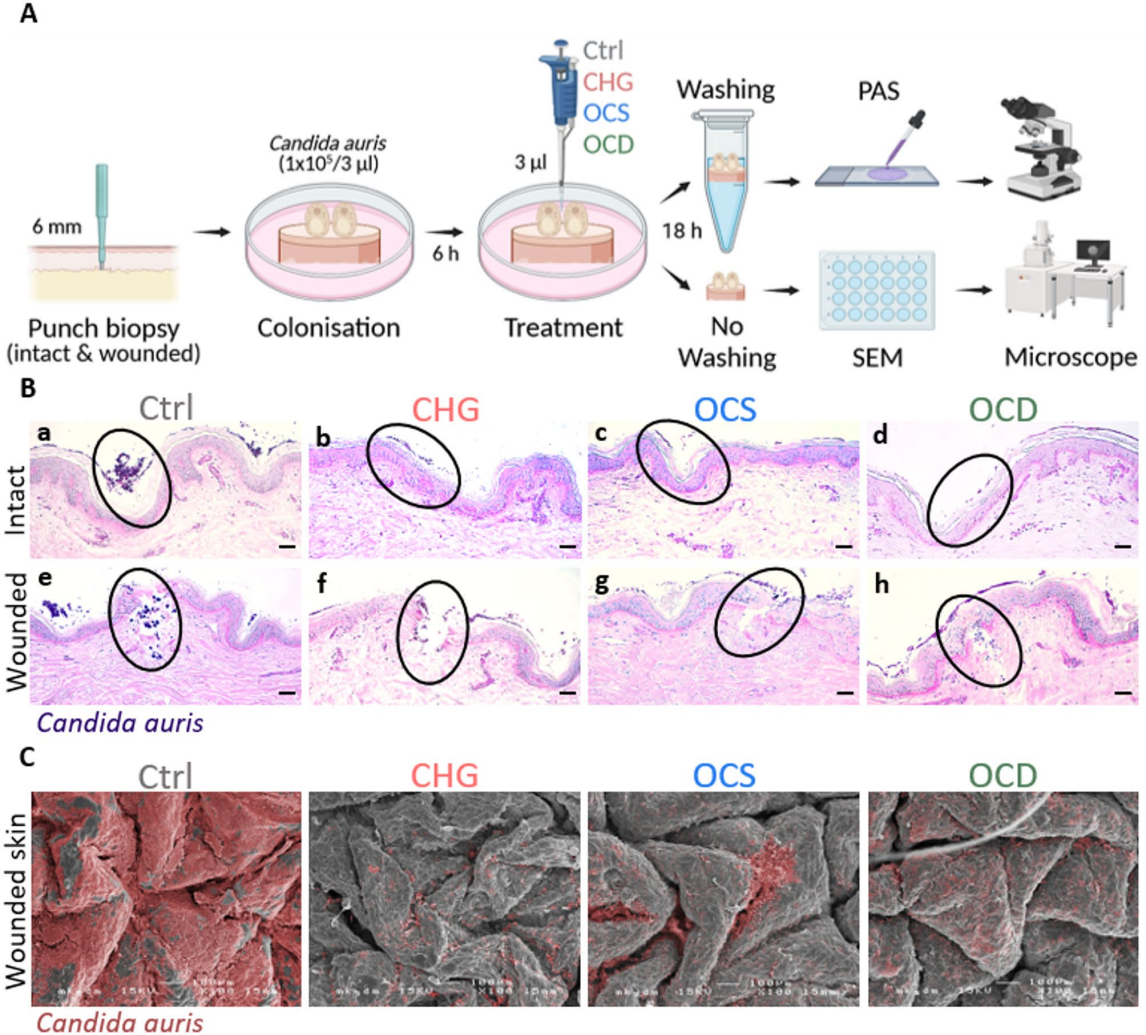
Topical antiseptic treatment markedly decreases *Candida auris* on intact and wounded human skin. (**A**) Workflow illustrating skin colonisation, application of antiseptics or control (Ctrl; PBS), sample preparation, and subsequent analysis (Created in BioRender. Cerbu, D. (2025) https://BioRender.com/qqoihjz). (**B**) Representative Periodic Acid-Schiff (PAS) staining images are depicted from one donor out of seven for both, intact as well as wounded (needled) skin. A reduction of fungal population and infiltration in ex vivo intact (a-d) and wounded (e–h) human skin following antiseptic treatment (CHG: chlorhexidine gluconate; OCS: octenisept; OCD: octeniderm), compared to the control is demonstrated. Scale bar: 50 µm. (**C**) Representative scanning electron microscopy (SEM) images are shown from one donor out of four. They reveal *Candida auris* biofilm formation on wounded ex vivo human skin and a reduced bioburden (pseudo-colored in red) after antiseptic treatment compared to the control. Magnification 1000x. Scale bar: 100 µm.

Freshly isolated abdominal and thigh skin was obtained from anonymous healthy adult donors (female/male, age range: 29–62 years, n = 7) after plastic surgery. Skin was disinfected pre-surgically, immediately cleaned with PBS after excision and either left untreated (“intact skin”) or subjected to needling (four passes; “wounded skin”) using a micro-needling pen (Winpok, China) with 12 needles measuring 2-millimetres (mm) in length^34^. Tissue biopsies of 6 mm in diameter were then obtained as punch biopsies (Brymill Cryogenic Systems, USA) from intact and wounded skin and transferred into 12-well culture plates (Techno Plastic Products, Switzerland). To each well 600 μl of Dulbecco’s Modified Eagle’s Medium (DMEM) was added, supplemented with 10% fetal bovine serum and 1% penicillin–streptomycin (all Thermo Fisher). A 3 µl suspension containing 1 × 10^5^ *C. auris* cells (1133/P/13R) was applied topically to each skin biopsy and culture plates were incubated at 37 °C in 5% CO_2_ for 6 h (Fig. 1A).

### Topical disinfection and culture conditions

After the incubation of *C. auris*-contaminated skin, each biopsy was treated topically with octenisept (OCS; 0.1% OCT aqueous solution), octeniderm (OCD; 0.1% OCT alcoholic solution, both Schülke & Mayr GmbH, Austria), 2% CHG (aqueous magistral formula, provided by a local pharmacy) or PBS (control). The treatment modalities included either a single (× 1) topical 3 μl drop of OCS, OCD, PBS, CHG or three (× 3) 3 μl drops of OCS and OCD. To prevent draining, a repeated application was carried out in which the liquids were administered in steps of 1.5 μl until the desired amount was obtained (Fig. 1A).

### Periodic acid-Schiff (PAS) staining and scanning electron microscopy (SEM) imaging

After cultivation for 18 h, biopsies were collected and fixed for i) PAS staining in 7.5% formaldehyde (SAV liquid production GmbH, Germany), and ii) SEM in Karnovsky’s fixative (2% paraformaldehyde, 2.5% glutaraldehyde in 0.1 M phosphate buffer pH 7.4; Morphisto, Germany) for at least 24 h.

Paraffin-embedding, deparaffinization and histological PAS staining were conducted in accordance with previously established protocols^34^, and the microscopy images were processed (brightness and contrast across the entire image) using ImageJ software (Version 1.54f.; Java 1.8.0_322; 64-bit; National Institute of Health; https://imagej.net/ij/). Skin biopsies were prepared for SEM as described previously and examined in a SEM (JSM 6310, Jeol Ltd, Japan) at an acceleration voltage of 15 kilovolts^80,81^ (Fig. 1A).

### Skin digestion and CFU quantification

**Fig. 2:**
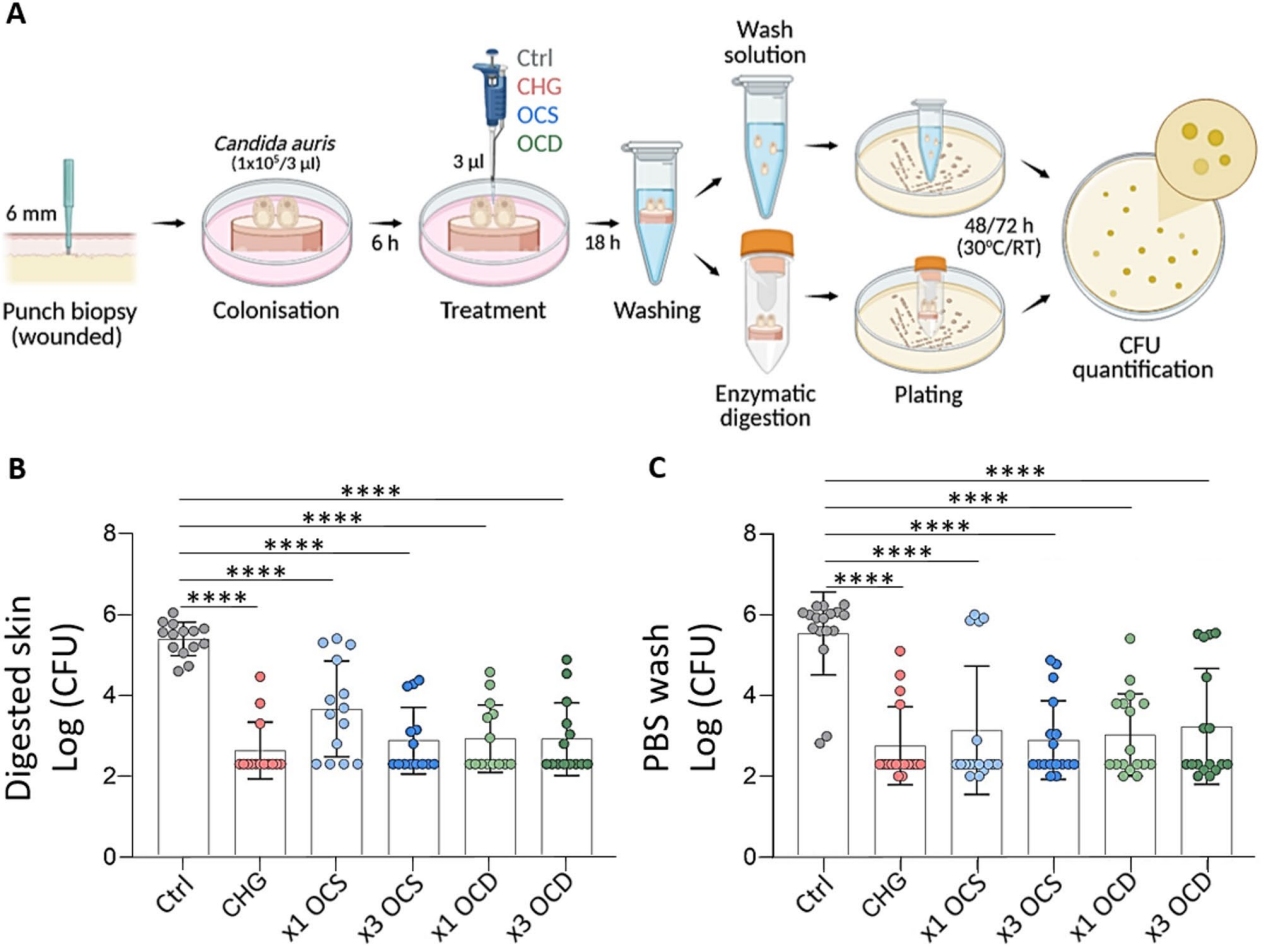
Antiseptic formulations significantly reduce *Candida auris* bioburden on wounded human skin. (**A**) Schematic workflow outlining the infection and treatment strategy, along with a washing step and enzymatic digestion of the skin, plating and quantification steps (Created in BioRender. Cerbu, D. (2025) https://BioRender.com/zvyhljd). Colony forming unit (CFU) counts on skin biopsies (**B**), and biopsy wash solution (**C**). *Candida auris* CFUs are displayed on a logarithmic scale, and the data was analyzed using GraphPad Prism (version 10.4.0.). One-way ANOVA, ****P < 0.0001, (n = 7). Each sample was tested in duplicates. Control (Ctrl): PBS; Antiseptics: CHG: chlorhexidine gluconate; OCS: octenisept; OCD: octeniderm; × 1: single topical application, × 3: triple topical application, RT: room temperature.

Contamination, treatment, and culture of skin biopsies were carried out as shown in Fig. 1A. After washing each biopsy in 1 ml of PBS, the wash solution was retained for CFU quantification. The washed biopsy was processed to quantify *C. auris* cells adhered to the skin surface and infiltrated through needle punctures. Thus, biopsies were cut with scissors and transferred to gentleMACS C-tubes (Miltenyi Biotec, Germany) for digestion (Fig. 2A). Aliquots of 500 μl of an enzymatic solution containing collagenase P (Roche, Switzerland) (1 mg/ml) were added to each tube and incubated (37 °C, 5% CO_2_) for approximately 2 h. Following enzymatic digestion, 1 ml of PBS was added, and skin samples were homogenized using the gentleMACS Octo Dissociator (Miltenyi Biotec). The homogenate was tenfold serially diluted up to a factor of 100. For quantification, 100 μl of each diluted sample was pipetted in duplicates on YPD plates. Likewise, 1 ml of PBS from washed biopsies was diluted by a factor of 100 and plated to control for viable *C. auris* cells potentially removed during the washing step. The plates were incubated at 30 °C for 48 h or at room temperature for 72 h for subsequent quantification. CFUs were calculated and plotted using GraphPad Prism (version 10.4.0; GraphPad Software; https://www.graphpad.com/) (Fig. 2A).

###  In vitro *C. auris* biofilm eradication

**Fig. 3:**
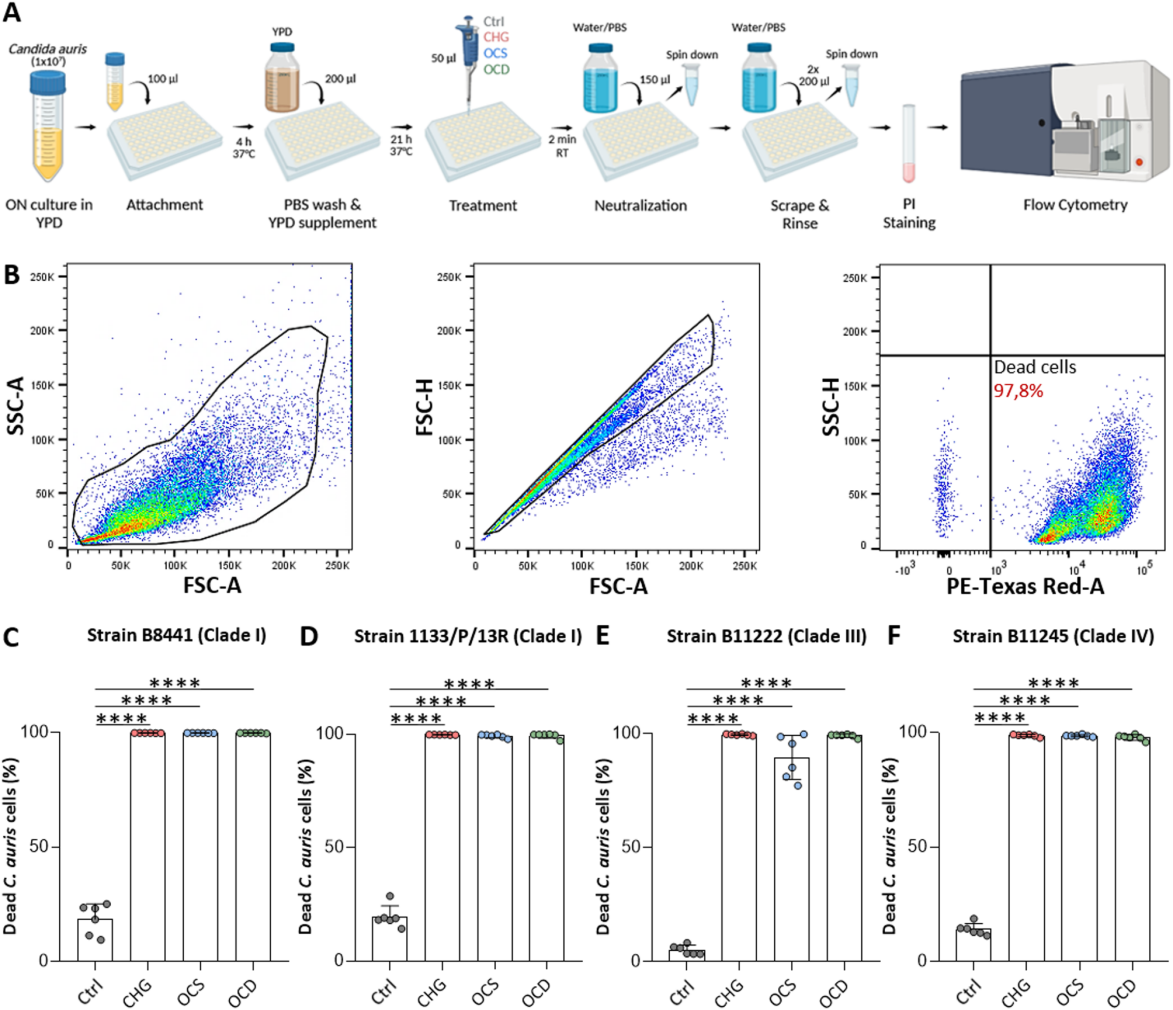
Antiseptic treatment eliminates *Candida auris* biofilms in vitro. (**A**) Workflow for the preparation and growth of *Candida auris* biofilm in vitro, treatment with antimicrobials (CHG: chlorhexidine gluconate; OCS: octenisept; OCD: octeniderm), and subsequent FACS analysis (Created in BioRender. Cerbu, D. (2025) https://BioRender.com/cxxxcyj). (**B**) A representative sample (OCS) was chosen to show the FACS gating strategy to identify propidium iodide (PI)-positive *Candida auris* cells. (**C**–**F**) Graphs display the percentage of *Candida auris* cells across different clades and strains killed after antiseptic treatment relative to the control Ctrl; PBS, as measured by flow cytometry. All analyses were performed using GraphPad Prism (version 10.4.0.). ANOVA for comparing multiple means to the control mean, ****P < 0.0001. Data from three independent experiments performed in duplicate/strain. ON: overnight; RT: room temperature; PI: propidium iodide.

A 100 μl suspension of *C. auris* clinical isolates (1133/P/13R, B8441, B11222, B11245) at a cell density of 1 × 10^7^ cells/ml in YPD medium was aliquoted into each well of a flat-bottom 96-well plate and incubated at 37 °C for 4 h to enable cell attachment. Afterwards, the culture medium was gently aspirated, and each well was washed with PBS. Subsequently, 200 μl of fresh YPD medium was added to promote biofilm growth for another 21 h at 37 °C. The next day, the biofilm was washed with PBS and visually assessed under a microscope (EVOS M5000 Invitrogen, Thermo Fisher Scientific, Germany). For treatments, 50 μl of OCS, OCD, 2% CHG or a control (PBS) was added to the respective wells in duplicates. After 2 min incubation, the reaction was neutralized by adding 150 μl of PBS (for PBS control wells) or water (for all other samples to prevent undesirable interactions) and the plate was immediately placed on ice. The contents of each well were transferred into 5 ml FACS tubes (Falcon, USA), centrifuged (5 min, 4800 × rpm) and the supernatant was carefully removed. Another 200 μl of PBS or water was added to the wells and the surface of each well was scraped to collect any remaining biofilm material. This process was repeated twice, followed by another centrifugation step. Cell suspensions were stained using propidium iodide (PI) Sigma-Aldrich, USA) diluted to a final concentration of 20 μg/ml in 0.5 mM EDTA in PBS. Stained cells were measured on a BD LSR Fortessa Cell Analyser (BD Biosciences) (Fig. 3A), and data was analysed using FlowJo software (Version 10.8.0; BD Life Sciences, https://www.flowjo.com/). Mean fluorescent intensity (MFI) of PE-TexasRed signals were exported (Fig. 3B).

### Statistical analyses

All statistical analyses were conducted using GraphPad Prism. The data were assessed for normality and analysed using one-way ANOVA with ****P < 0.0001; (n = 7). The sample size was sufficient to detect significant differences among groups, taking into account ethical considerations and the limited availability of fresh human material.

## Results

### OCT-based antiseptics efficiently reduce* C*.* auris* on and in ex vivo human skin

To determine the antifungal activity of applied substances, we established a workflow (Fig. 1A). Topical antimicrobial treatment of intact and wounded skin biopsies previously exposed to *C. auris* (Fig. 1B) revealed substantial differences in surface adhesion and infection patterns. Consistent with other reports^28,34,77,82^, *C. auris* is unable to penetrate or breach the intact skin barrier. Fungal cells adhere to and grow on the outermost skin surface, predominantly colonising within skin grooves or microfolds^30^. Hence, in PBS control samples, *C. auris* displayed a substantial growth on the surface, particularly in skin grooves (Fig. 1B, a; highlighted with a black outline), whereas treatment with CHG (Fig. 1B, b), OCS (Fig. 1B, c) or OCD (Fig. 1B, d) led to a marked reduction in viable fungal cells, especially in densely colonised regions. Following skin barrier disruption via needling, *C. auris* gains the ability to enter the dermal compartment (Fig. 1B, e). In contrast to the control, topical application of CHG (Fig. 1B, f), OCS (Fig. 1B, g) and OCD (Fig. 1B, h) led to a pronounced reduction of *C. auris* in this setting. Overall, PAS staining results emphasize the distinct *C. auris* infiltration patterns of skin biopsies treated with antiseptics relative to PBS, therefore underscoring a therapeutic potential in limiting fungal bioburden on and in human skin tissue.

In addition to histological analyses, we evaluated the impact of CHG, OCS and OCD on *C. auris* colonisation on wounded skin biopsies using SEM (Fig. 1C). In the control group, a prominent fungal presence was identified, characterized by a smooth matrix covering the entire skin surface (pseudo-coloured in red for better visualisation). Remarkably, biopsies treated with CHG, OCS and OCD exhibited considerably less *C. auris* cells on the skin surface, indicating that these antiseptics efficiently reduce the pathogen.

### OCS and OCD significantly reduce* C*.* auris* bioburden in skin compartments

Given the limitations of obtaining quantitative data from histology and SEM assessments, our next objective was to evaluate the efficacy of each antiseptic on and in contaminated ex vivo human wounded skin through CFU quantification. CFU counts from digested skin biopsies are shown in Fig. 2B, while CFU counts from the wash solution of the same biopsies are depicted in Fig. 2C. All tested antimicrobials not only significantly reduced topical *C. auris* bioburden, but also prevented from tissue invasion (Fig. 2B). These findings were reflected by a consistently low CFU count across most replicates when using OCS or OCD, comparable to CHG, all of them significantly lower than PBS control. Therefore, all antiseptics targeted the skin-tropic fungal pathogen on and in human tissue and seem relevant not only therapeutically but also for infection control measures.

### OCS and OCD significantly eradicate* C*.* auris* biofilm in vitro

We next investigated the antifungal efficacy of CHG, OCS and OCD against an adhesive *C. auris* biofilm^23,30^, commonly known for its reduced susceptibility to antimicrobial agents. Thus, we used four well described *C. auris* isolates from diverse geographic origins (clades I, III and IV), each exhibiting distinct antifungal susceptibilities as well as varying biofilm formation and adhesion properties^27,34,78,83–89^. The antifungal activity of CHG and the OCT-based formulations was evaluated using a standardized biofilm formation assay similar to the recently described method published by Singh and colleagues^90^. In each experiment, microscopic analysis consistently confirmed biofilm formation, revealing multi-layered structures with high cell density - hallmarks of mature biofilms. As reported previously, *C. auris* biofilms generally reach maturity after approximately 24 h under standard incubation conditions^91^. To assess cell viability within the biofilms, PI staining was performed and cell death was quantified by flow cytometry (Fig. 3A). A representative OCS-treated sample was selected to demonstrate the flow cytometry gating strategy used to identify PI-positive, non-viable *C. auris* cells (Fig. 3B). Strikingly, all isolates treated with antiseptics showed an almost complete eradication of *C. auris* biofilms and strong antifungal efficacy (up to 99.9%) when compared to the PBS control (Fig. 3C–F). Hence, quantification of fungal clearance highlighted the potent antifungal activity of tested antiseptic formulations even in a biofilm model.

## Discussion

Due to the alarming global rise of multidrug-resistant *C. auris* causing difficult-to-control nosocomial outbreaks, adjusted hygiene protocols are urgently needed^4^. Next to potent agents for environmental disinfection, effective antiseptics are mandatory to prevent *C. auris*-associated infections and to minimize the risk of skin-to-skin transmission among individuals in healthcare settings^9^ Skin colonisation by *C. auris* represents a major risk factor for subsequent invasive infections causing significant morbidity and mortality due to already limited antifungal treatment options^9^. Although being frequently reported in the bloodstream, *C. auris* infections have been also linked to severe wound, catheter-tip and intra-abdominal infections^92–95^. In that regard, the study presented herein evaluates for the first time the efficacy of commercial OCT-based antiseptics in reducing *C. auris* colonisation on ex vivo human skin.

Although animal models have significantly contributed to a better understanding of skin physiology and immune defence responses, their anatomical and physiological differences generally limit their human applicability^96–101^. Consequently, ex vivo primary human skin models emerged as a more appropriate alternative for studying skin-pathogen interactions in humans^96,99,102^, including investigations on the mode of action of antiseptics^103^. In this study, we employed our recently established ex vivo* C. auris* infection model using intact and wounded human skin^34^.

Ex vivo human skin biopsies remain viable in culture media for several days^104^, offering a unique model to study immediate early events related to adhesion of skin pathogens or for evaluating the effectiveness of antimicrobials on previously contaminated skin^77^. In our first series of experiments (Figs. 1, 2), we used a well characterized multidrug-resistant *C. auris* isolate corresponding to clade I^78,105–107^. It was directly applied onto intact and wounded human skin biopsies to determine the antifungal potential of two approved pharmaceuticals, which are based on the antiseptic molecule OCT: i) OCS, an aqueous wound and mucous membrane antiseptic, and ii) OCD, an alcoholic skin antiseptic. By subjecting human skin samples to needling, a standardized procedure that creates defined holes reaching to the dermal compartment, we aimed to mimic clinical conditions where the natural skin barrier is compromised^34^. Of note, every breach of the skin barrier such as medical device implantation, total parenteral nutrition, catheter placement or surgery can serve as a portal of *C. auris* entry to the blood stream and deeper tissues^22^. Thus, exploring effective antimicrobial strategies are key to combat *C. auris* colonisation on both intact and wounded skin. In that regard, bathing of patients with a 2% CHG solution or treatment with 2% CHG wipes represents a common approach for decolonizing patient’ skin in many clinical settings worldwide, however, with reported limited effectiveness against *C. auris*^18,108^.

The results presented herein confirm our previous findings that *C. auris* can adhere to and grow on intact human skin, preferentially colonising skin grooves (Fig. 1B, a)^34^ without invading the tissue^77^. We show that topically infected intact skin biopsies treated with CHG (Fig. 1B, b), OCS (Fig. 1B, c), and OCD (Fig. 1B, d) displayed a substantial decrease in fungal bioburden. Following physical disruption of the skin barrier, *C. auris* readily infiltrated deeper tissue layers and the high bioburden indicates a robust fungal presence within the deeper skin microenvironment, suggesting unimpeded adherence and proliferation in PBS-treated controls (Fig. 1B, e). Conversely, topical application of CHG (Fig. 1B, f), OCS (Fig. 1B, g) and OCD (Fig. 1B, h) exhibited a reduced occurrence of *C. auris* also in needling holes. The effect of CHG, OCS and OCD becomes more apparent by SEM imaging (Fig. 1C) of wounded skin, demonstrating markedly diminished coating with *C. auris* when compared to control samples, where *C. auris* cells are covering the entire skin surface.

By quantifying *C. auris* CFUs in washed and enzymatically digested samples from wounded skin (Fig. 2B, C), we demonstrated that application of OCS and OCD significantly reduced fungal bioburden both on (Fig. 2B) and within (Fig. 2C) human skin, equal to CHG. Overall, both OCT-based antiseptic treatment regimen proved to be highly effective against *C. auris*, regardless if applied once or even three times. These findings collectively indicate that OCT is potent in clearing *C. auris* on compromised human skin, thus offering a further effective option for decontamination of colonised patients and consequently, probably reduce the risk of a subsequent systemic infection.

Virulence and resistance of *C. auris* to antifungal agents are clade-specific^109^. For our next approach, we selected isolates from clade I (Asia)^78,84,110^, clade III (African), and clade IV (South Amercian)^86^, reported to have multidrug-resistant characteristics^109,111^. Moreover, biofilm formation also varies among clades and likely contributes to prolonged *C. auris* persistence on human skin and tolerance to antimicrobials^22^. In that regard, we further showed in an in vitro* C. auris* biofilm model adapted from Singh et al.^90^ (Fig. 3A) that CHG, OCS and OCD were highly effective in eliminating *C. auris* cells from different clades (Fig. 3C–F). This confirms the potent and unspecific mode of action of the tested compounds to disintegrate biofilms to 100% when compared to the control.

### Limitations and future directions

While our *ex vivo* human skin model can yield valuable insights into antiseptic efficacy by preserving native tissue architecture and resident immune cell populations, it does not capture systemic components such as circulating immune cells, vascular dynamics, and hormonal or organ-level interactions, all of which are likely to impact OCT’s antifungal activity in vivo. In addition, donor-to-donor variabilities, including differences in skin microbiome compositions, age, sex and other host factors could alter both baseline susceptibility and the residual activity of OCT-based antiseptics against *C. auris*. Natural shedding during nosocomial outbreaks as well as mechanical impact such as skin wiping using wash mitts could additionally influence *C. auris* reduction on patients next to pure antiseptic efficacy of the product itself. To address some of these limitations, future studies should i) employ *in vivo* animal models to evaluate the roles of systemic immunity, blood circulation, metabolism, and long-term recolonization dynamics; ii) establish standardized high-bioburden *ex vivo* challenge models under more rigorous colonization conditions; and iii) include real-life clinical measures considering factors like mechanical forces during wiping, which were not replicated in the present ex vivo human skin model. Particularly, clinical trials would be crucial to validate the effectiveness of OCT-based commercial products, especially those intended for whole body decolonisation during nosocomial *C. auris* outbreaks.

In summary, our results show that CHG, OCS and OCD equally efficient reduce *C. auris* colonization of both intact and wounded human skin. Compared to CHG, OCT-based products may offer a promising option for nosocomial decolonisation, given their previously reported long-term residual effect, high safety profile (extremely low allergic potential with no reported risk for anaphylactic reactions) as well as no induction of resistance to the antiseptic OCT itself or even cross-resistance to other antifungal agents. Altogether, these findings reinforce key antifungal stewardship efforts and most importantly, may contribute to improved patient safety.

## Data Availability

The data used and/or analysed during the current study is available in anonymous format (according to data protection policy in the ethics agreement) from the corresponding author on reasonable request.
